# Deconstructing pheromone-mediated behavior one layer at a time

**DOI:** 10.1186/1741-7007-12-33

**Published:** 2014-05-29

**Authors:** Gabriela Sánchez-Andrade, Darren W Logan

**Affiliations:** 1 Wellcome Trust Sanger Institute, Hinxton, Cambridge CB10 1SA, UK

## Abstract

The vomeronasal organ, a sensory structure within the nasal cavity of most tetrapods, detects pheromones that influence socio-sexual behavior. It has two neuronal layers, each patterned by distinct receptor sub-families coupled to different G-proteins. Work recently published in this journal found female mice with one layer genetically inactivated are deficient in a surprisingly wide range of reproductive behaviors, providing new insights into how the nose can influence the brain.

See research article: http://www.biomedcentral.com/1741-7007/12/31

## Commentary

All animals secrete or excrete complex chemical signatures into the external environment: in sweat, tears, urine, feces or in scent marks from specialized exocrine glands. These have the potential to provide information on the identity, sex, age, fitness and threat afforded by the animal that deposited them. Consequently, many species have evolved to use these for inter-individual communication. One of the sensory subsystems that detect such chemicals is the vomeronasal organ (VNO), a highly specialized neuroepithelium found in the base of the nose of most tetrapods. The VNO is responsible for detecting intra-specific and inter-specific chemical signals, known as pheromones and kairomones, respectively, that initiate innate behaviors [1].

**Figure 1:**
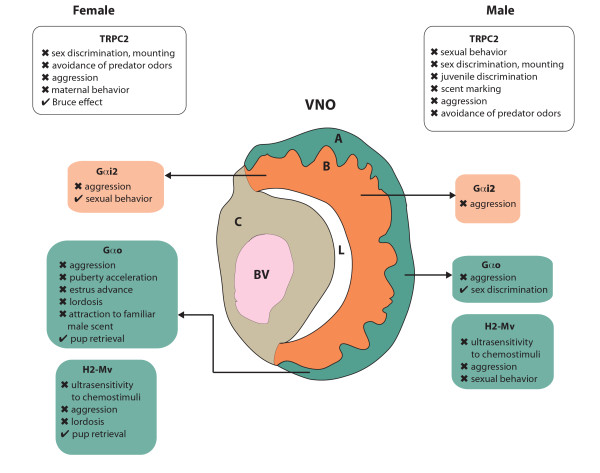
**The mouse vomeronasal organ (VNO) and its influence on behavior.** In the center, a coronal section of half a VNO is represented. A, apical layer of sensory epithelium; B, basal layer of sensory epithelium; BV, blood vessel; C, cavernous tissue; L, lumen. Behaviors affected (crosses) and not affected (ticks) by ablation of genes that pattern the VNO in male (right) and female (left) mice. Each box is shaded to indicate the layer of the VNO that the gene patterns. TRPC2 (white) is expressed in both layers.

The VNO epithelium is organized into two cellular layers (Figure 1). Neurons in the apical layer express a receptor from the V1R family and the Gαi2 G-protein, which are together thought to initiate signal transduction after the receptor binds a semiochemical ligand. Those in the basal layer express V2R receptors and the Gαo G-protein, which are likely to fulfill a similar function [2]. Neurons in both layers express a member of the transient receptor potential family of ion channels, TRPC2, a downstream component of both signal transduction pathways [3]. V1R/Gαi2 and V2R/Gαo neurons project to different aspects of the accessory olfactory bulb in the brain; thus, the two layers of the VNO may transmit very different types of information. While significant progress has been made towards defining the source and chemical characteristics of the ligands that bind V1R and V2R receptors in mice [2], the sufficiency and necessity of each neuronal layer in instructing differential behavior are still largely unknown. This is due, in part, to the complex genetic engineering involved in generating animals that have one layer entirely and exclusively inactivated [4]. In this journal, Oboti *et al*. report an extremely comprehensive analysis of sexual and reproductive behavior in mice with a non-functional V2R/Gαo layer [5]. This new work, along with a companion study of aggressive behavior in the same mutant line [6], marks the first step towards deconstructing the behavioral logic encoded within the layers of the VNO.

## Behavioral consequences of layer specific inactivation

Gαi2 and Gαo have been independently ablated in mice previously [7,8]. Although both mutant lines do display abnormal behavior, the G-proteins are expressed in other tissues so the precise role of the VNO in the behavioral phenotype could not be determined. To resolve this, Oboti and colleagues applied the Cre-Lox recombinase system to delete Gαo only in cells that express olfactory marker protein (OMP). Although these genes are co-expressed in some other sensory neurons in the nose, albeit at lower levels, only those in the basal VNO layer show a significant reduction in Gαo expression [6].

When tested in a battery of assays, a remarkably large range of behavioral and physiological deficiencies are observed in these mice (Figure 1). For example, male urine is unable to induce estrus in adult mutant females (a phenomenon known as the Whitten effect) while juveniles fail to undergo puberty acceleration in response to male urinary cues (the Vandenbergh effect). Adult female mice also fail to adopt a characteristic mating stance when exposed to male suitors, and are unable to detect and remember male-specific non-volatile individuality cues [5]. In addition, both mutant males and postpartum mothers display severely curtailed aggressive responses to intruders [6]. These phenotypes are consistent with the loss of acute pheromone signaling through V2Rs (coupled to Gαo) in the basal layer of the VNO. However, similar behavioral abnormalities have been reported in *Trpc2-/-* mice, which have both VNO layers inactivated [2]. Does this imply the apical layer of the VNO has no role in mediating aggressive or sexual behaviors? We consider this unlikely, as there are aggression-promoting semiochemicals in male urine that activate the V1R/Gαi2 layer exclusively [9]. It is possible that deactivating one layer could have an indirect effect on the function of the other. However, mice lacking basal layer function do display some behavioral differences to those with the whole VNO genetically inactivated, suggesting the Gαi2 layer is at least partly functional. A more feasible explanation may be that parallel signaling through both layers is required to instruct certain behaviors. Although initially segregated, evidence suggests that circuits downstream of V1R and V2R neurons converge deeper in the brain where synergistic activation could be necessary to generate a behavioral response.

## Is the VNO more than a pheromone sensor?

Interrupting signal transduction in the VNO appears to have consequences beyond acute pheromone detection. *Trpc2-/-* mice have a reduced number of neurons in the VNO, suggesting its activity is important for cellular maintenance [3]. The basal VNO layer is also partially degenerated in conditional Gαo mutant mice, while the apical layer remains unaffected [6]. What phenotypic impact might a reduction in the number of neurons in the basal layer have on the mice? Curiously, group-housed conditional Gαo mutant females have severely disrupted estrus cycles, suggesting the basal layer of the VNO influences reproductive physiology independent of male pheromone signaling [5]. The mechanism through which this occurs is unclear. Gonadotropin releasing hormone (GnRH) neurons migrate from the VNO to the forebrain during early development, where they control the release of reproductive hormones. It is therefore tempting to speculate that a degenerated basal layer could interfere with GnRH neuron migration or function. However, circulating reproductive hormone levels and ovary morphology appear normal in adult mice and *Trpc2-/-* females, with degeneration in both VNO layers, are not reported to have irregular estrus cycles [5]. This unexpected phenotype may hint at a more complex relationship between the VNO and reproductive physiology than previously thought.

## Future directions in understanding VNO function

Producing mice with other subsets of VNO neurons inactivated is becoming ever easier due to technological advances in genetic engineering [4]. Indeed, another recent study used a sophisticated chromosome engineering approach to further functionally subdivide the V2R/Gαo layer [10]. Members of the *H2-Mv* cluster, a family of non-classical class I major histocompatibility complex genes, are expressed in approximately half of the cells in the basal layer of the VNO. When these genes are deleted the pheromone sensitivity of the neurons that express them is significantly decreased, but not entirely lost [10]. This results in both sexual and aggressive deficits even though two-thirds of the VNO remains unperturbed, further supporting a synergistic model of pheromone signaling.

Ultimately, the systematic gene targeting of over 500 V1R and V2R genes will be necessary to reveal the full extent of the behavioral logic encoded within the molecular organization of the VNO, though, even in the era of large-scale knockout mouse programs, this will be a significant undertaking [4]. In the meantime, increasingly sensitive transcriptomic analyses are likely to reveal genes that pattern the olfactory neurons of the nose in smaller and smaller sub-divisions. Conditionally deleting these in the VNO of mice should continue to provide novel insights into the pheromone-mediated behaviors of mammals.
